# Cold acclimation can specifically inhibit chlorophyll biosynthesis in young leaves of Pakchoi

**DOI:** 10.1186/s12870-021-02954-2

**Published:** 2021-04-10

**Authors:** Huiyu Wang, Zhubo Li, Lingyun Yuan, Hefang Zhou, Xilin Hou, Tongkun Liu

**Affiliations:** 1 State Key Laboratory of Crop Genetics and Germplasm Enhancement, Key Laboratory of Biology and Genetic Improvement of Horticultural Crops in East China, Ministry of Agriculture and Rural Affairs of the P.R. China, Engineering Research Center of Germplasm Enhancement and Utilization of Horticultural Crops, Nanjing Agricultural University, Nanjing, 210095 China; 2 College of Horticulture, Vegetable Genetics and Breeding Laboratory, Anhui Agricultural University, Hefei, 230036 China; 3 Huainan Agricultural Science Institute, Huainan, 232001 China

**Keywords:** 5-aminolevulinic acid (ALA), *BrFLU*, Chlorophyll biosynthesis, Cold acclimation, Leaf color conversion, Pakchoi

## Abstract

**Background:**

Leaf color is an important trait in breeding of leafy vegetables. Y-05, a pakchoi (*Brassica rapa *ssp*. chinensis*) cultivar, displays yellow inner (YIN) and green outer leaves (GOU) after cold acclimation. However, the mechanism of this special phenotype remains elusive.

**Results:**

We assumed that the yellow leaf phenotype of Y-05 maybe caused by low chlorophyll content. Pigments measurements and transmission electron microscopy (TEM) analysis showed that the yellow phenotype is closely related with decreased chlorophyll content and undeveloped thylakoids in chloroplast. Transcriptomes and metabolomes sequencing were next performed on YIN and GOU. The transcriptomes data showed that 4887 differentially expressed genes (DEGs) between the YIN and GOU leaves were mostly enriched in the chloroplast- and chlorophyll-related categories, indicating that the chlorophyll biosynthesis is mainly affected during cold acclimation. Together with metabolomes data, the inhibition of chlorophyll biosynthesis is contributed by blocked 5-aminolevulinic acid (ALA) synthesis in yellow inner leaves, which is further verified by complementary and inhibitory experiments of ALA. Furthermore, we found that the blocked ALA is closely associated with increased *BrFLU* expression, which is indirectly altered by cold acclimation. In BrFLU-silenced pakchoi Y-05, cold-acclimated leaves still showed green phenotype and higher chlorophyll content compared with control, meaning silencing of *BrFLU* can rescue the leaf yellowing induced by cold acclimation.

**Conclusions:**

Our findings suggested that cold acclimation can indirectly promote the expression of *BrFLU* in inner leaves of Y-05 to block ALA synthesis, resulting in decreased chlorophyll content and leaf yellowing. This study revealed the underlying mechanisms of leaves color change in cold-acclimated Y-05.

**Supplementary Information:**

The online version contains supplementary material available at 10.1186/s12870-021-02954-2.

## Background

Leaf color is determined by pigments, which mainly includes chlorophyll, carotenoids, and anthocyanins [1, 2]. Plant leaf color change are always caused by a variety of factors and can be achieved in many ways [3], such as, chlorophyll biosynthesis and breakdown [4], chloroplast assembly [5], and signal transduction in plant disease resistance [6]. For many plant species, the leaf color is green, which is given by chlorophyll. Chlorophyll, the essential cofactors for photosynthesis [2], has been studied for many years [7, 8]. Chlorophyll enables the plants to use sunlight via photosynthesis. Maintaining chlorophyll normal state and level is essential for photosynthetic efficiency and carbon fixation [9], then directly influence plant growth and development [10, 11].

Numerous Chl-deficient species, also called leaf color mutations, which exhibited significant changes in chlorophyll synthesis or degradation mechanism and various phenotypes in different species, were regarded as suitable materials for exploring the mechanism of chlorophyll biosynthesis [12–15]. For instance, in rice, there were some missense mutations occurred in conserved amino acid of ChlD and ChlI in *chl1* and *chl9* mutants, respectively. *chl1* and *chl9* mutants showed poorly stacked grana and underdevelopment chloroplasts [16]. The other color mutant *F03–06* in *Arabidopsis*, controlled by recessive mononuclear gene *At5g54810*, the gene-silenced plants exhibited similar phenotype with *F03–06*, yellow leaves with mottled veins, the plants are stunted and growth slowly [17]. A stably inherited *Brassica* plant etiolated mutation (*pem*) with DNA sequence variation in the promoter of *Bra024218*, showed retarded chloroplast development, decreased chlorophyll content and reduced photosynthetic capacity [18]. The chlorophyll (Chl)-deficient mutant *pylm* of pakchoi had yellow leaves with reduced total Chl content, loose grana lamellae structure and few thylakoid stacks, lower photosynthetic activity and photochemical conversion efficiency, which are caused by the block of Chl *a* production and down-regulation of genes related with Chl biosynthesis [19]. Mutants with loss-of-function REDUCED CHLOROPLAST COVERAGE (REC) protein, which is homologous with tetratricopeptide repeat (TPR) protein, showed lower chlorophyll contents and smaller chloroplast compartment size compared to wild type in *Arabidopsis* [20]. Pentatricopeptide repeat (PPR) protein, which motif is assumed to have evolved from a TPR proteins, is associated with various functions including temperature-sensitive chlorosis [21, 22]. Among these leaf color mutations, which exhibits normal or near-normal leaf color at room temperature, but exhibits significant change in leaf color at low temperature, are identified as low temperature-sensitive type [23]. Diverse analytical methods based on multiple omics databases were selected to comprehensively explore chlorophyll biosynthesis involved color conversion in low temperature-sensitive type, including tea (*Camellia sinensis* L.) [24], rice (*Oryza sativa* L*.*) [21], wucai (*Brassica campestris* L.) [9] at molecular or protein level. However, the mechanism of leaf color change response to low temperature is not totally understood.

Chlorophyll is synthesized in the chloroplast and distributed on the thylakoid membranes in chloroplast [25]. The general process of chlorophyll biosynthesis is composed of three main steps: (1) formation of 5-aminolevulinic acid (ALA) from glutamate, (2) formation of protoporphyrin IX from ALA, and (3) formation of chlorophyll from protoporphyrin IX [26]. ALA has been attracted attention as a key precursor that participated in biosynthesis of chlorophyll and plants greening [27, 28]. Exogenous application of ALA has been shown to increase the chlorophyll content of plants [29–31]. Previous reports indicated that ALA is formed from Glu in the C_5_ pathway consisting of two steps [32]. First, under the effects of glutamyl-tRNA reductase (GluTR), the Glu-tRNA converts to Glu-1-semialdehyde (GSA). Second, GSA converts to ALA by the effects of GSA-2,1-aminomutase (GSA-AM) [33]. GluTR and GSA-AM are encoded by the nuclear *HEMA* and *GSA* genes, respectively. Among the enzymes of ALA synthesis, GluTR is regarded as the main rate-limiting step [34]. Fluorescent in blue light (FLU), a tetratricopeptide repeat (TPR) protein, is the best-known negative regulator of ALA synthesis. Previous researches suggested that FLU protein can interact with the C-terminal segment of GluTR to inactivate ALA synthesis, resulting in reduced chlorophyll content and etiolated seedling [35, 36]. In FLU-overexpression (FLUOE) *Arabidopsis*, FLUOE lines show pale leaves under medium light and yellow-green leaves under low light owning to decreased chlorophyll contents [37]. In addition, another factor regulating GluTR is GluTR-binding protein (GBP). GBP has been shown to interact with the N-terminal region of GluTR to protect GluTR from degradation, maintaining adequate ALA synthesis [34].

Pakchoi (*Brassica rapa *ssp*. chinensis*), a subspecies of Chinese cabbage, is a widely consumed vegetable in Asia, especially in China. Since the main edible organ of pakchoi is leaf, leaf color is an important trait in breeding. Y-05 is a special pakchoi cultivar which displays green leaves grown under normal condition but displays yellow inner (YIN) and green outer (GOU) leaves after cold acclimation. Due to its special phenotype, Y-05 is considered as an interesting and valuable material for cold-acclimated leaf color change research. To date, there has been little research on the leaf color change of Y-05 response to cold acclimation, and its color conversion mechanism remains elusive. Here, we aimed to characterize the changed leaf color of pakchoi Y-05 at the physiological, cellular and molecular levels. Our findings contribute to the understanding of cold acclimated Y-05 displaying yellow inner and green outer leaves. These findings also enrich our understanding of the mechanism of leaf color change.

## Results

### The inner leaves of cold-acclimated Y-05 exhibit decreased chlorophyll content and undeveloped thylakoids

**Fig. 1:**
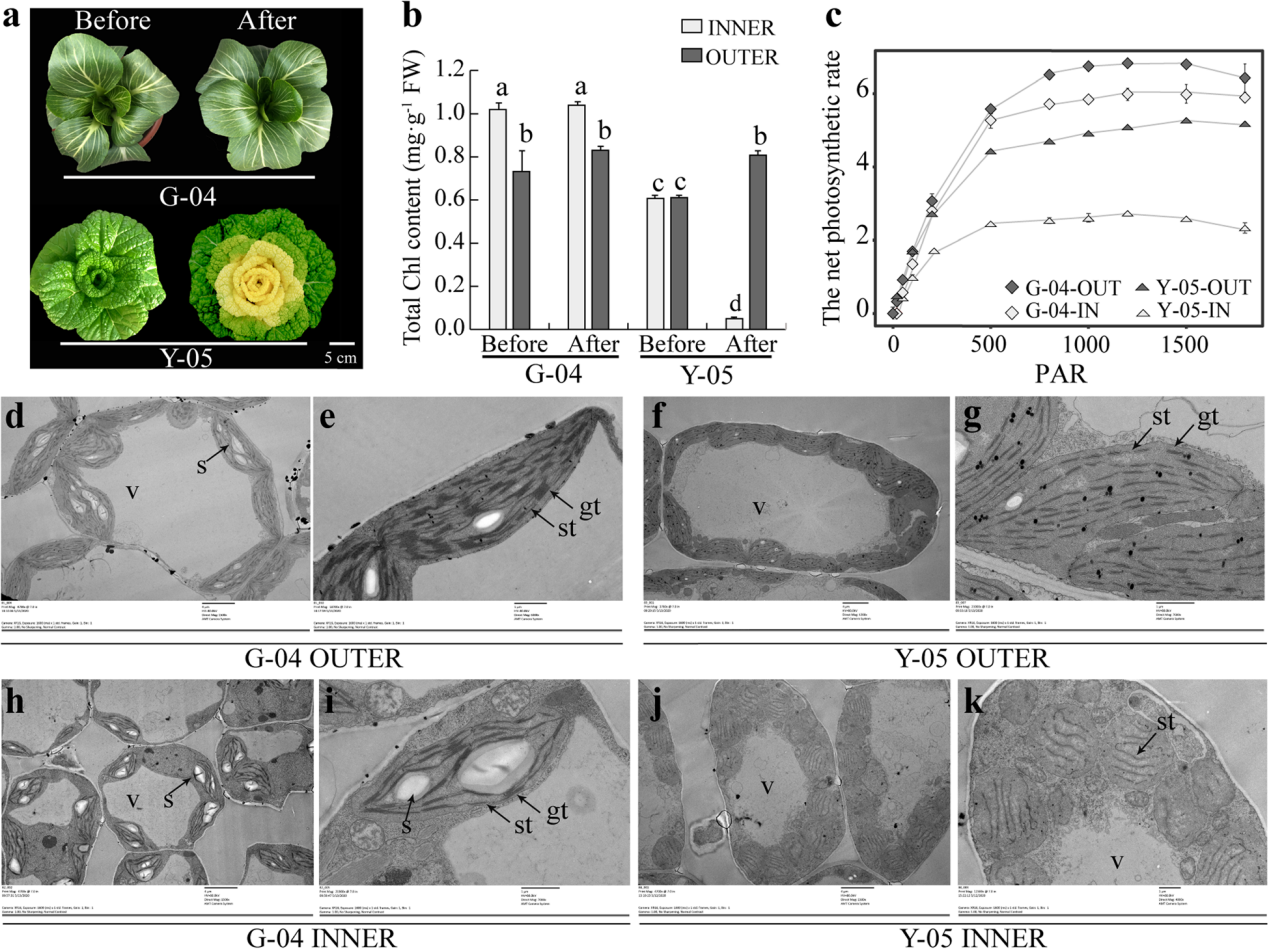
The inner leaves of cold-acclimated Y-05 exhibit decreased chlorophyll content and undeveloped thylakoids. **a** The phenotype of G-04 and Y-05 before and after cold acclimation. For cold acclimation, two-month old Y-05 plants were grown 3 weeks at 4 °C, and then return to 23 °C for continues grown. Before, before cold acclimation. After, after cold acclimation. Bar = 5 cm. **b** The total chlorophyll content of outer and inner leaves from Y-05 and G-04 before and after cold acclimation. **c** The net photosynthetic rate (P_n_) of outer and inner leaves from Y-05 and G-04 after cold acclimation. PAR, photosynthetic active radiation. Three individual plants of each cultivar were quantified, and the total chlorophyll content and P_n_ were measured three times. Error bars represent SE (±SE, *n* = 3). Different letters indicated statistically significant differences at the level of *p* < 0.05. **d**-**k** Chloroplast ultrastructure of outer and inner leaves from G-04 and Y-05 after cold acclimation. v = vacuole, s = starch grains, gt = granum thylakoids, st = stroma thylakoids. In the Fig. 1d, f, h, j, Bar = 4 μm. In the Fig. 1e, g, i, k, Bar = 1 μm

Compared with green leaves of pakchoi cultivar G-04, Y-05 displays yellow inner and green outer leaves after cold acclimation (Fig. 1a). Hence, we took the pigments measurement between G-04 and Y-05 at different growth conditions and periods. Interestingly, all of them show decreased contents in yellow inner leaves compared with leaves of G-04 and green outer leaves of Y-05 (Fig. 1b, Fig. S1). Generally, the content of carotenoids, xanthophylls and anthocyanin increased rather than decreased, which could cause yellow leaf phenotype [1, 2]. So, we proposed that the yellow leaf phenotype of Y-05 maybe caused by low chlorophyll content.

Chlorophyll is the main pigment for photosynthesis in plants and located in the thylakoid membrane of chloroplast [38]. To verify our hypothesis, we checked the photosynthetic capacity (P_n_) of outer and inner leaves between cold-acclimated Y-05 and G-04. As our expected, both outer and inner leaves of cold-acclimated Y-05 possess significantly decreased net photosynthetic rate compared with the outer and inner leaves of cold-acclimated G-04 respectively (Fig. 1c).

Next, we further studied the chloroplast ultrastructure of inner and outer leaves from cold-acclimated G-04 and Y-05 by transmission electron microscopy (TEM). Observation of the cold-acclimated G-04 showed lots of mature chloroplasts and thick granum-thylakoids in outer leaves (Fig. 1d, e), and developing chloroplasts and thin granum-thylakoids in inner leaves (Fig. 1h, i). In cold-acclimated Y-05, the green outer leaves show mature chloroplasts but thinner grana stacks compared with outer leaves of G-04 (Fig. 1f, g). However, the yellow inner leaves of Y-05 display undeveloped chloroplasts and almost disappeared granum-thylakoids (Fig. 1j, k). Together, we suggested that the yellow inner leaves of Y-05 are caused by decreased chlorophyll content and undeveloped thylakoids which maybe induced by cold acclimation.

### Transcriptomes and metabolomes of cold-acclimated Y-05

**Fig. 2:**
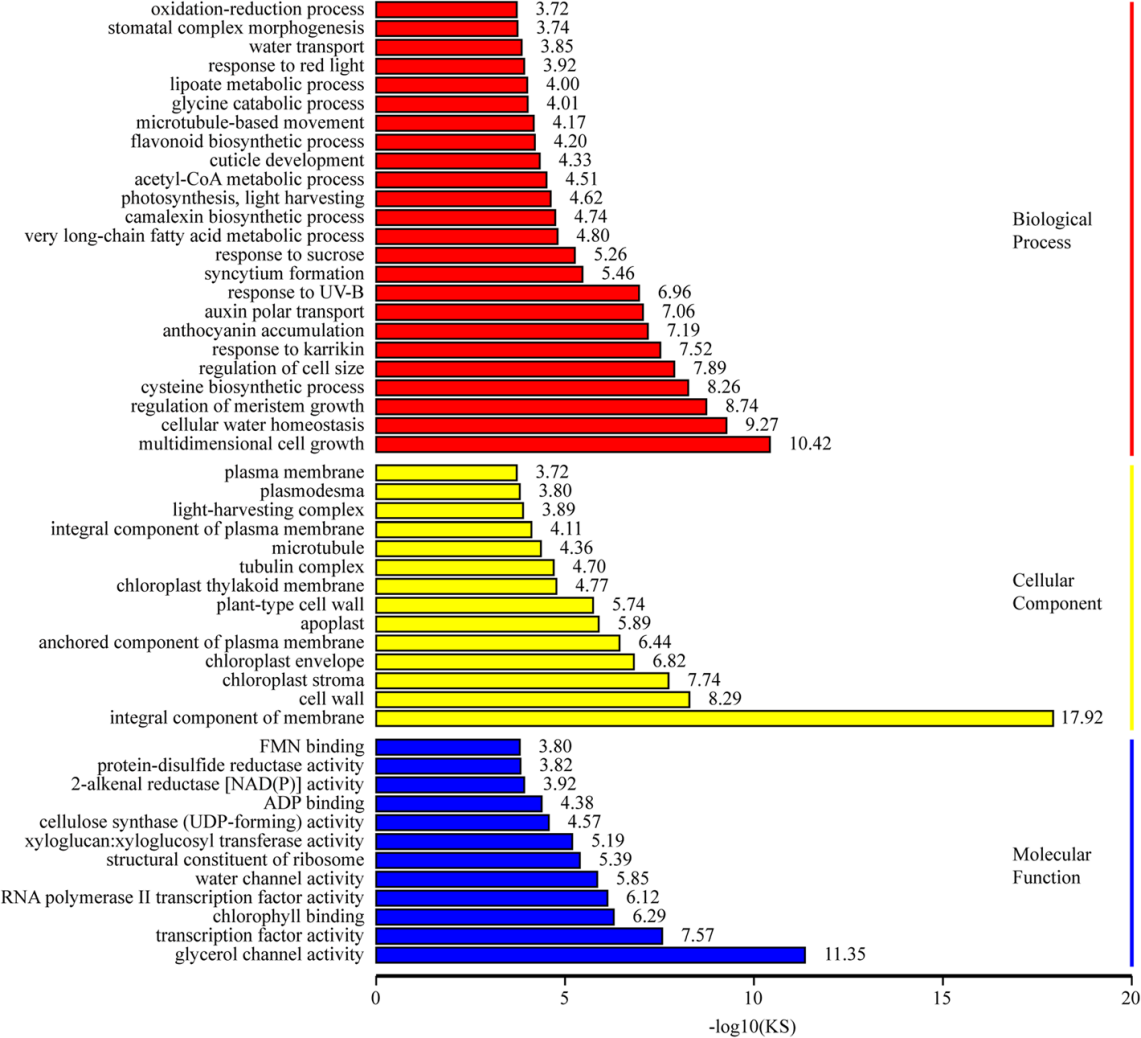
The enriched GO pathway of the differentially expressed genes (DEGs) between TOU and TIN of pak choi Y-05. -log10 (KS) represents the statistical significance of GO term. The bigger of -log10 (KS), the more significant enriched

**Fig. 3:**
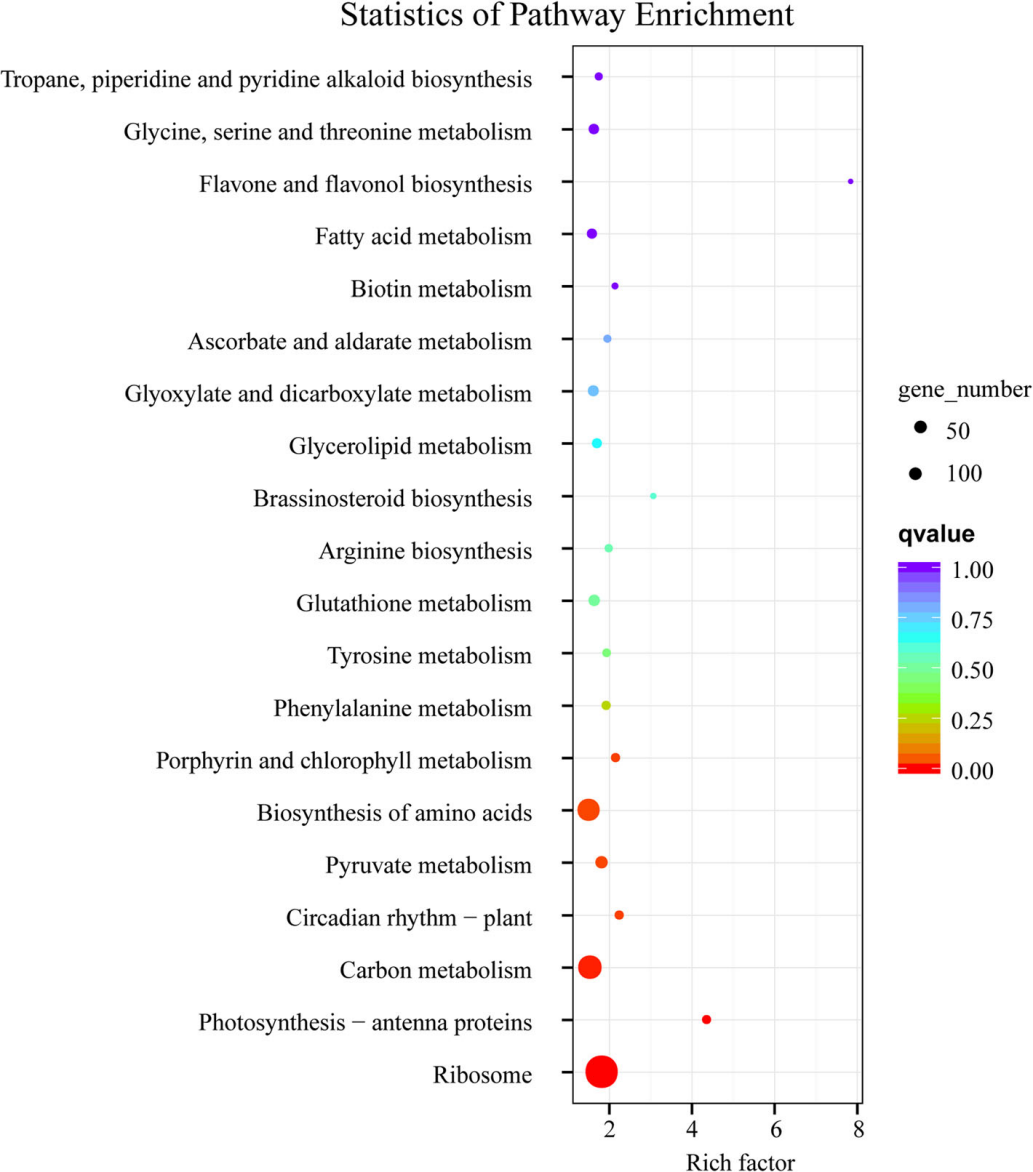
The top 20 enriched KEGG pathway of the differentially expressed genes (DEGs) between TOU and TIN of pak choi Y-05. Each point represents a KEGG pathway, ordinate represents pathway name, and abscissa represents the enrichment factor. The larger the enrichment factors, the more significant the enrichment level of DEGs are showed. The color of the circle represents *q*-value, lower *q*-value means the more reliable results. And the size of the circle represents the number of genes enriched in the pathway

To explore the molecular mechanisms of leaf color change in Y-05 induced by cold acclimation, the transcriptomes of green outer leaves (TOU) and yellow inner leaves (TIN) were conducted (Table S1–4). For both the TOU and TIN samples, three independent biological replicates were set up. The high correlation coefficient indicates a strong linear relationship between biological duplications (Fig. S2a). Totally, 4887 differentially expressed genes (DEGs) were identified between the YIN and GOU, 2239 genes were down-regulated and 2648 genes were up-regulated in the YIN compared with GOU (Fig. S2b, Table S2). Gene Ontology (GO) analysis revealed that the integral component of membrance and chloroplast-related categories were overrepresented in cellular component (Fig. 2, Table S3). Kyoto Encyclopedia of Genes and Genomes (KEGG) pathway analysis revealed significant changes in the ‘Photosynthesis-antenna proteins’(ko00196), ‘circadian rhythm-plant’(ko04712) and ‘Porphyrin and chlorophyll metabolism’(ko00860) pathway with rich factor > 2 (Fig. 3, Table S4).

**Fig. 4:**
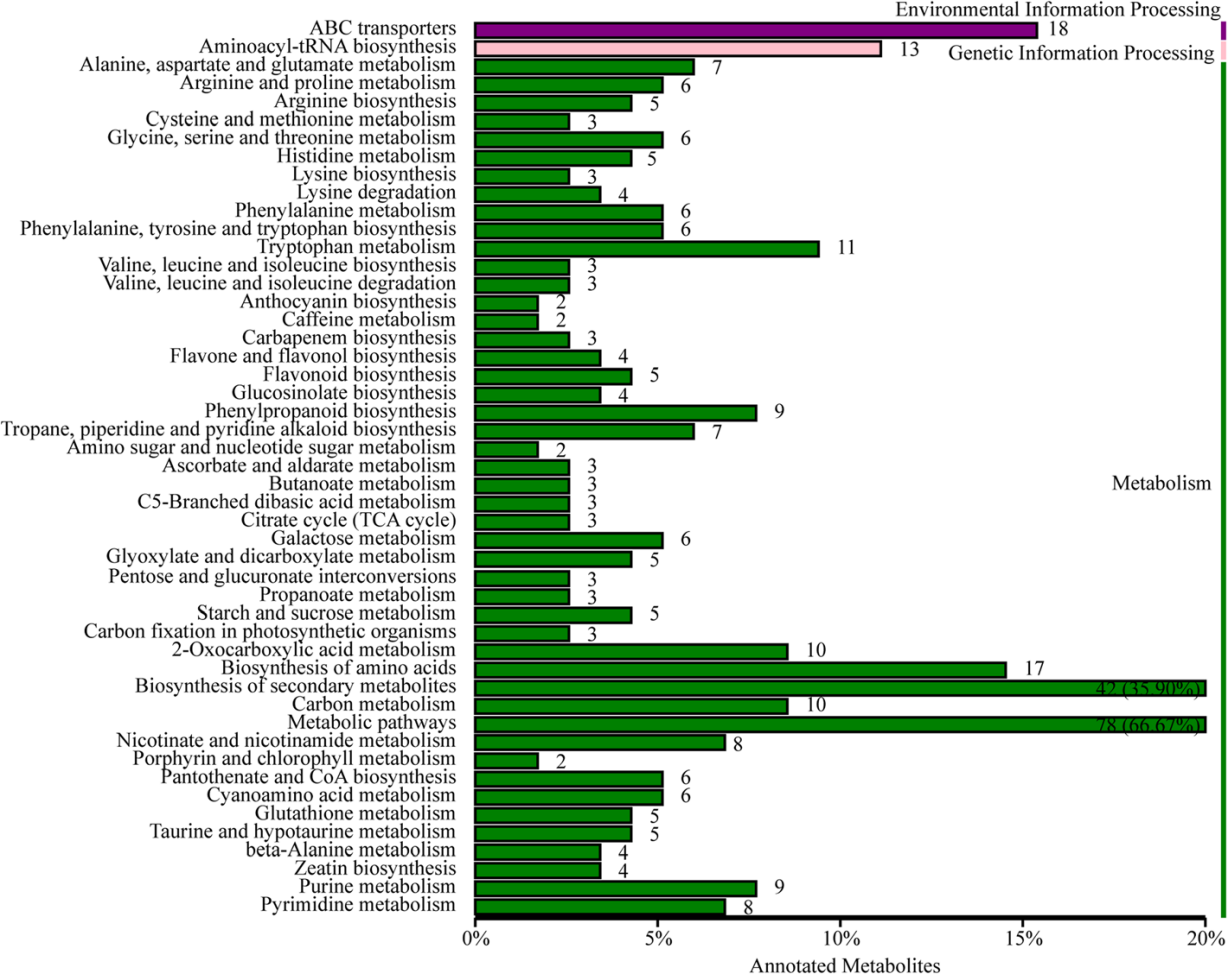
The enriched KEGG pathway of the differentially expressed metabolites (DEMs) between inner-yellow (MIN) and outer-green (MOU) leaves of pakchoi Y-05. Short time-series expression miner (STEM) was used to analyze the metabolites expression pattern. The number of DEMs in each profile was labeled above the frame. The bar represents the proportion metabolites in each profile of the total annotated metabolites

For exploring the differences in the composition of metabolites in inner-yellow (MIN) and outer-green (MOU) leaves of Y-05, the non-targeted metabolomes analysis was performed. Repeatability and correlation analysis between MOU and MIN was assessed to prove data reliability (Fig. S3a). In total, 372 differentially expressed metabolites (DEMs) were identified. One hundred sixty-two metabolites were up-accumulated, and 210 metabolites were down-accumulated in MIN (Fig. S3b, Table S5). Next, we analyzed the DEMs between MIN and MOU. KEGG pathway analysis revealed that most metabolites are mainly enriched in the ‘metabolic pathways’ and ‘biosynthesis of secondary metabolites’ pathway (Fig. 4). Interestingly, the ‘Porphyrin and chlorophyll metabolism’ pathway was also found (Fig. 4), which consistent with our previous results that chlorophyll content changes in Y-05. From the metabolomes data, we found two metabolites involved in chlorophyll biosynthesis, 5-Aminolevulinate (ALA, meta_22) and L-Glutamic acid (meta_277), were down-regulated and up-regulated in MIN compared with MOU respectively (Table S6), meaning that the conversion from L-Glutamic acid to ALA is blocked in inner leaves. Together, we proposed that the decreased chlorophyll content in inner leaves of Y-05 is associated with the inhibition of ALA synthesis.

### ALA content is critical for the leaf color conversion of pakchoi Y-05

**Fig. 5:**
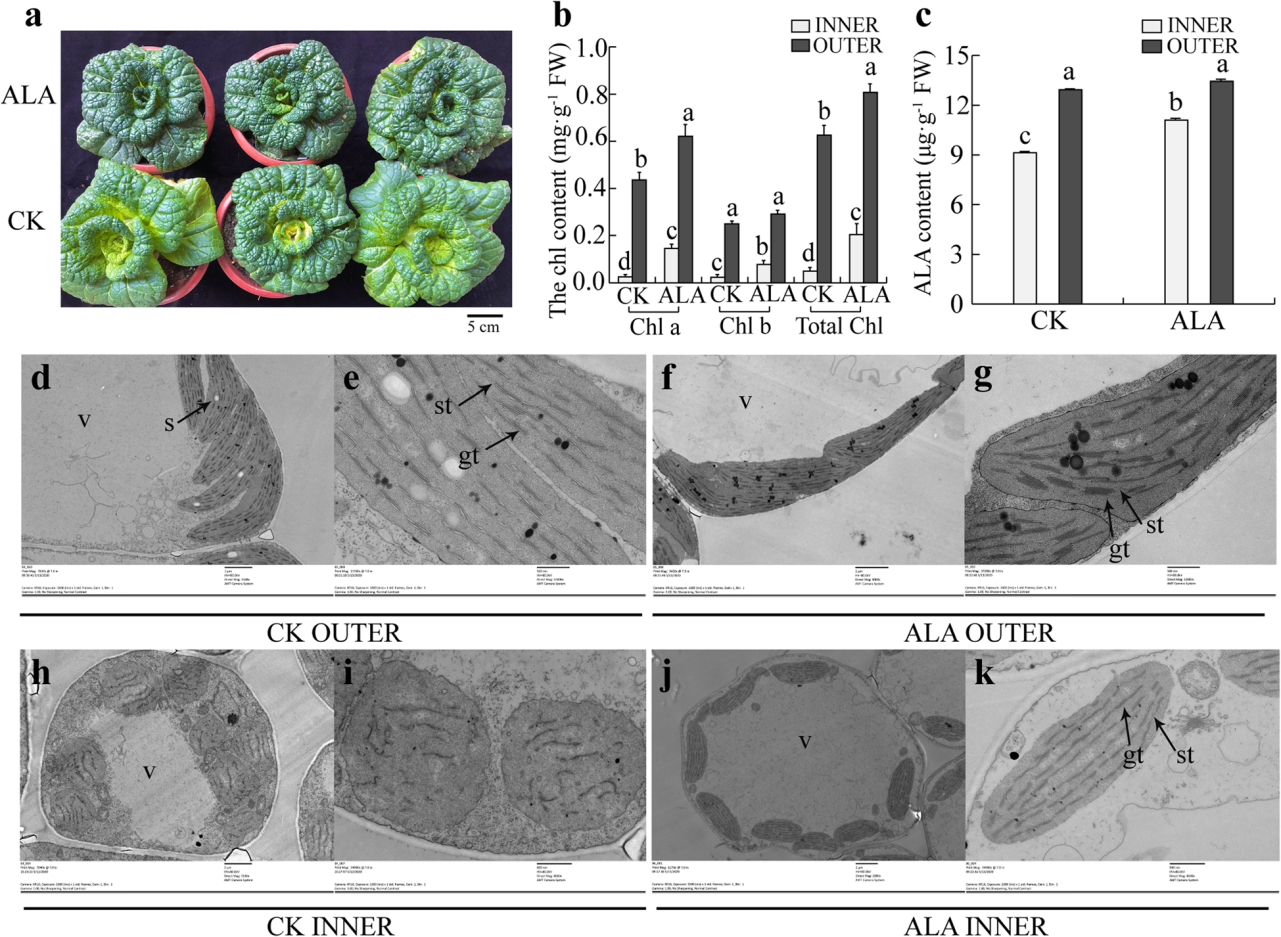
Exogenous application of ALA can rescue the yellow leaves phenotype in cold-acclimated Y-05. **a** The leaf phenotype of ALA-treated plants compared with CK plants. ALA, cold-acclimated Y-05 sprayed by 1 mM ALA. CK, cold-acclimated Y-05 sprayed by water as control. Bar = 5 cm. **b** The chlorophyll content of ALA-treated plants was higher than CK plants. Chlorophyll a, Chlorophyll b and total chlorophyll abbreviated as Chl a, Chl b, Total Chl, respectively. **c** The ALA content of ALA-treatment plants was higher than CK plants. Three individual plants of each cultivar were quantified, and the chlorophyll and ALA content were measured three times. Error bars represent SE (±SE, n = 3). Different letters indicated statistically significant differences at the level of *p* < 0.05. **d**-**k** Observation of chloroplast ultrastructure showed that ALA-treated plants possessed mature chloroplasts and granum thylakoids. v = vacuole, gt = granum thylakoids, st = stroma thylakoids. In the Fig. 4d, f, h, j, bar = 2 μm; in the Fig. 4e, g, bar = 500 nm; in the Fig. 4i, k, bar = 800 nm

Based on the metabolomes data (Table S5 and S6), the inhibition of ALA synthesis in inner leaves of cold-acclimated Y-05 may result in decreased chlorophyll content and leaf yellowing. To verify our hypothesis, 1 mM ALA was immediately sprayed on leaves of Y-05 after cold acclimation when both outer and inner leaves are still green. The cold-acclimated Y-05 plants sprayed by water were used as control. As our expected, the leaf yellowing is inhibited in ALA-treated inner leaves, while control plants display yellow inner leaves (Fig. 5a). Consistent with the phenotype, the chlorophyll and ALA content showed significantly increased in ALA-treated Y-05 (Fig. 5b, c). Further, we observed the ultrastructure of chloroplasts in ALA-treated and control leaves. The chloroplasts in outer leaves of ALA-treated plants possess thicker granum-thylakoids compared with control plants (Fig. 5d-g). Meanwhile, compared with the undeveloped chloroplasts in inner leaves of control, the inner leaves of ALA-treated plants possess mature chloroplasts and granum-thylakoids (Fig. 5h-k). Together, these results suggested that exogenous application of ALA can rescue the yellow phenotype of Y-05 induced by cold acclimation.

**Fig. 6:**
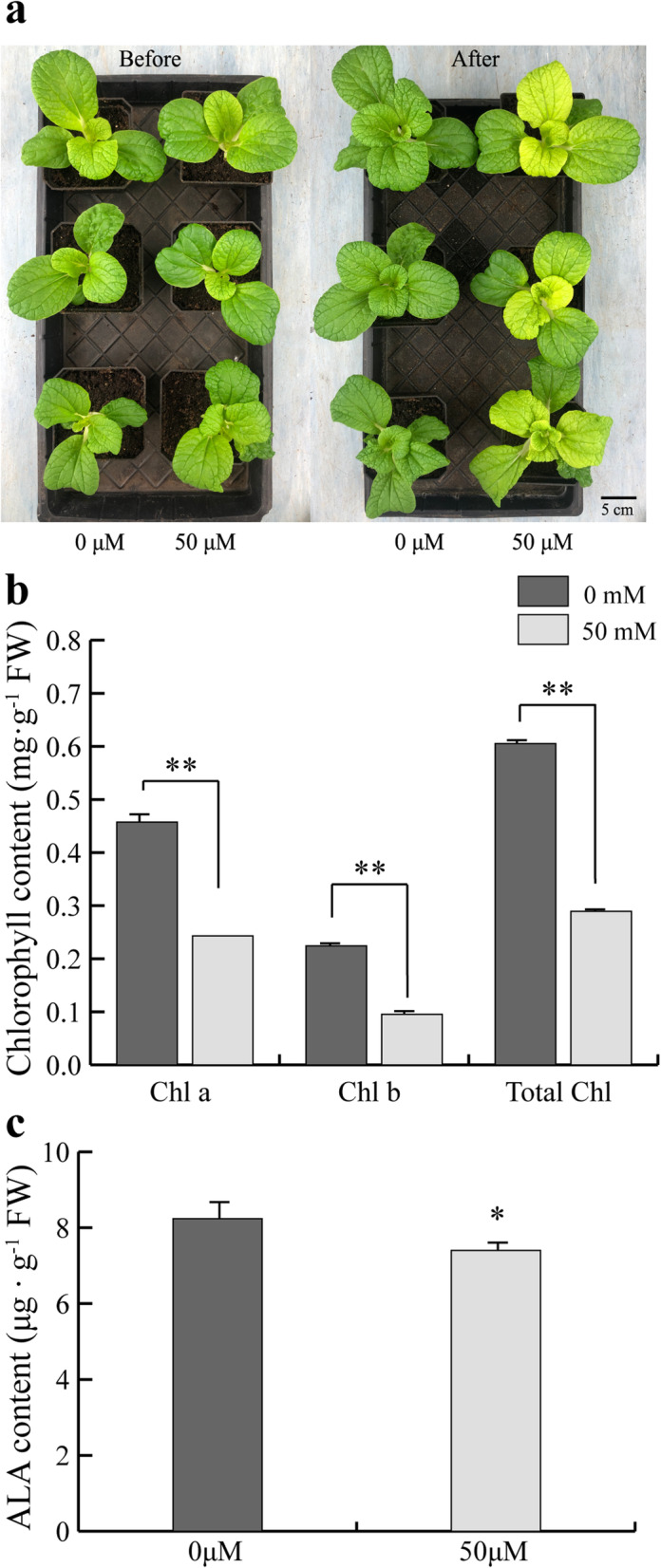
Gabaculine-treated Y-05 seedlings show yellow leaves phenotype without cold acclimation. **a** The gabaculine-treated plants showed yellow phenotype without cold-acclimation. Before, plants before treated with gabaculine. After, plants after treated with gabaculine. 0 μM and 50 μM represented CK plants and gabaculine-treated plants, respectively. Bar = 5 cm. **b** The inner leaves of gabaculine-treated plants showed low chlorophyll content compared with CK plants. **c** The inner leaves of gabaculine-treated plants showed low ALA content compared with CK plants. Three individual plants of each cultivar were quantified, and the chlorophyll and ALA content were measured three times. Error bars represent SE (±SE, n = 3). * represents *p* < 0.05, ** represents *p* < 0.01

Next, to further investigate the role of ALA in leaf color conversion of Y-05, gabaculine (3-amino 2,3-dihydrobenzoic acid), an inhibitor of ALA biosynthesis by inactivating GSA-AT activity [39, 40], was used to irrigate one-month-old Y-05 seedlings. The Y-05 seedlings watered by water were used as control. After treatment, the inner leaves of gabaculine-treated Y-05 seedlings display yellow phenotype without cold acclimation, while the control plants still show green inner leaves (Fig. 6a). Consistent with the phenotypes, the chlorophyll and ALA content in inner leaves decreased in gabaculine-treated plants compared with control plants (Fig. 6b, c). These findings further verified that the inhibition of ALA biosynthesis is the key in leaf yellowing of Y-05. Together with the above results, we suggested that the content of ALA is critical for the leaf color conversion of Y-05.

### The upregulation of *BrFLU* is critical for ALA inhibition in cold-acclimated Y-05

Above data suggested that ALA biosynthesis is blocked in yellow inner leaves of cold-acclimated Y-05. During chlorophyll synthesis process, the conversion of L-Glutamic acid to ALA is the rate-limiting step which is controlled by three positive regulators, *GBP*, *HEMA* and *GSA*, and one negative regulator *FLU* [34, 41]. To deeply study the molecular mechanism of ALA inhibition in YIN of cold-acclimated Y-05, we firstly studied the homologous genes expressions of *GBP*, *HEMA* and *GSA* between YIN and GOU leaves. The transcription data showed that the expression of *BrHEMA1* and *BrGSA1* are significantly increased in YIN compared with GOU leaves (Fig. S4, Table S7). Since *HEMA* and *GSA* are positive regulators of ALA biosynthesis, the ALA inhibition in YIN leaves should be not associated with increased *BrHEMA1* and *BrGSA1* expression. As for *BrGBP*, which showed no significant changes in expression level between YIN and GOU, also should not be the reason for blocked ALA (Fig. S4, Table S7). Previous studies indicated that FLU, a negative regulator of ALA biosynthesis, interacts with the C-terminal of GluTR to inactivate ALA synthesis [37, 42]. Interestingly, BraA05003715 (*BrFLU*), the homologous gene of Arabidopsis *FLU* in pakchoi, shows 7.4-fold high expression in YIN compared with GOU (Fig. S4, Table S7), which is consistent with decreased ALA content. Hence, *BrFLU* was selected as candidate gene of ALA inhibition in yellow inner leaves for next research.

**Fig. 7:**
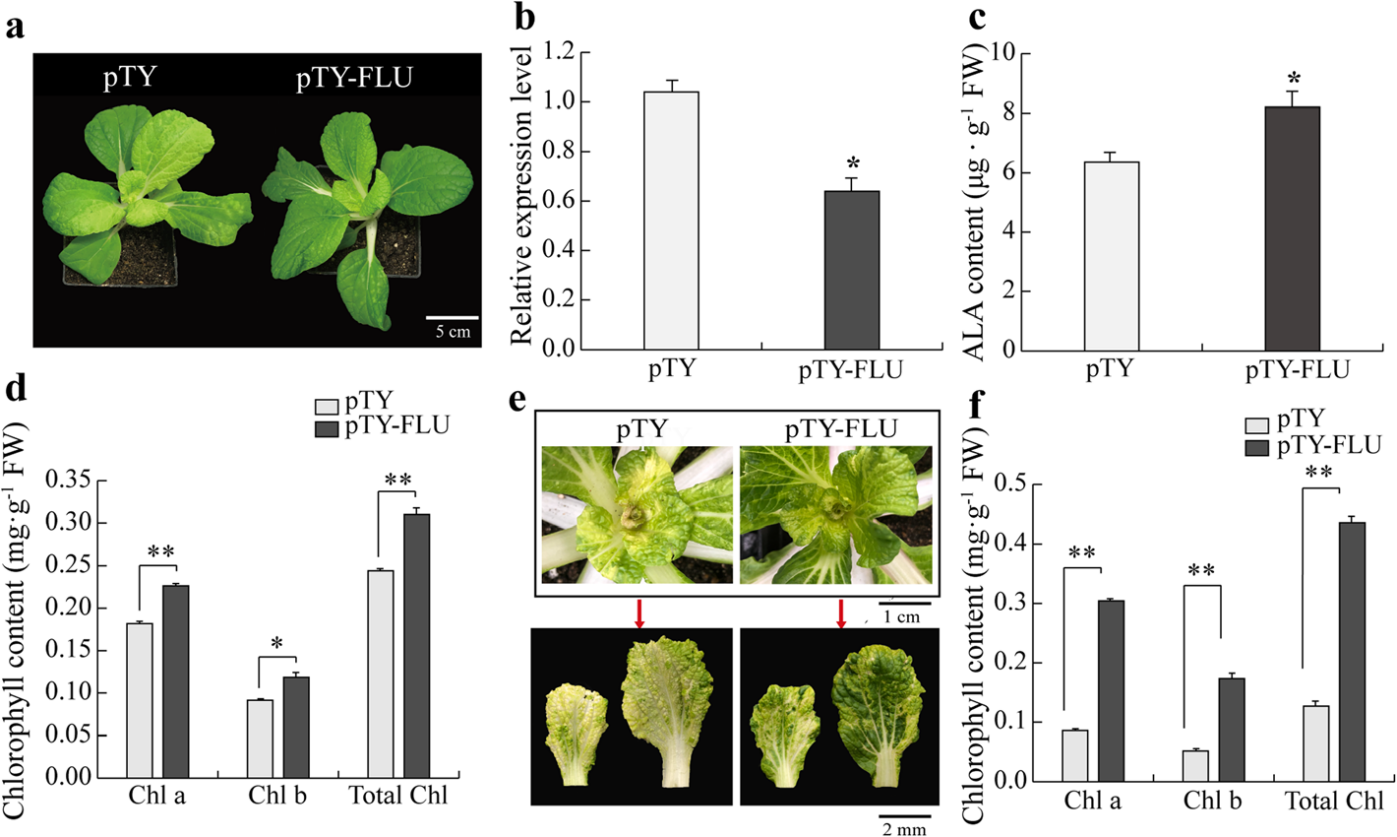
Silencing of *BrFLU* in Y-05 is critical for ALA biosynthesis. **a** The phenotype of pTY plants (CK) and pTY-FLU (BrFLU-silenced) plants. Bar = 5 cm. **b** The relative expression level of *BrFLU* significantly decreased in pTY-FLU plants. The inner leaves were selected to confirm the *BrFLU* expression. **c** The pTY-FLU plants showed higher ALA content than pTY plants. **d** The pTY-FLU plants showed higher chlorophyll content than pTY plants. **e** The phenotype of pTY and pTY-FLU plants after cold acclimation. Bar = 1 cm or 2 mm. **f** The pTY-FLU plants showed higher chlorophyll content than pTY plants after cold acclimation. Chlorophyll a, Chlorophyll b and total chlorophyll abbreviated as Chl a, Chl b, Total Chl, respectively. Three individual plants of each cultivar were quantified, and the chlorophyll content was measured three times. Error bars represent SE (±SE, n = 3). * represents *p* < 0.05, ** represents *p* < 0.01

To study the role of *BrFLU* in leaf color conversion of Y-05, the BrFLU-silenced line (pTY-FLU) of Y-05 was conducted (Fig. 7a, b). The Y-05 plants injected with pTY empty vector were used as control (pTY). Compared with control plants, the silenced plants showed more chlorophyll and ALA content (Fig. 7c, d), suggesting that low *BrFLU* level makes positive contribution to ALA and chlorophyll biosynthesis in Y-05. Further, if the *BrFLU* upregulation induced by cold acclimation is closely related with ALA inhibition and decreased chlorophyll content in Y-05 inner leaves, silencing of *BrFLU* in cold-acclimated Y-05 should increase chlorophyll content and rescue the yellow leaves phenotype, at least in part. To verify our hypothesis, pTY and pTY-FLU lines were treated with 0 °C for 3 weeks. Excitingly, pTY-FLU plants showed green phenotype and higher chlorophyll content compared with control plants (Fig. 7e, f), suggesting that the *BrFLU* upregulation is closely related with leaf color conversion of Y-05. Together, these data suggested that the upregulation of *BrFLU* induced by cold acclimation is critical for ALA inhibition, finally resulting in decreased chlorophyll content and yellow inner leaves of Y-05.

Furthermore, to explore why *BrFLU* is specially upregulated in cold-acclimated Y-05, we first compared the *BrFLU* open reading frame (ORF) sequences between Y-05 and other stay-green pakchoi varieties (G-04, WTC, 2Q, LY, MET, SZQ). Although many single nucleotide polymorphisms in *BrFLU* were detected between Y-05 and other pakchoi varieties (Fig. S5), the BrFLU amino acid sequences were found no significant different between Y-05 and other varieties (Fig. S6). Since motif appearing or missing in promoter will affect gene expression level, we then analyzed the promoters of *BrFLU* in Y-05 and other pakchoi varieties. Unfortunately, we did not found any motif specific appearing or missing in promoter of *BrFLU* from Y-05 (Fig. S7). Taken together, we proposed that the specific upregulation of *BrFLU* in Y-05 is induced by its upstream regulator which responds to cold acclimation.

## Discussion

Leaf color is an important trait in vegetable breeding and marketing. However, previous studies on the mechanism of leaf color conversion were mainly focus on trees and ornamental plants [43, 44]. Although some researches on leaf color conversion were carried out in crops such as rice, maize and wheat [21, 24], there are few studies on vegetables. Here, we studied the mechanism of leaf color conversion in pakchoi. To explore the color conversion mechanism of special phenotype of Y-05 (Fig. 1a), we first measured the pigment contents between yellow and green leaves, including chlorophyll, carotenoids, xanthophyll and anthocyanin. Results showed all pigments decreased in the yellow inner leaves of Y-05 (Fig. S1). Previously, many studies found that leaf color conversion is usually accompanied by senescence [45]. For instance, the chlorophyll was continuously degraded whereas the carotenoids was partial retained during the process of leaf senescence, was thought to be the reason of leaf color change in *Ginkgo biloba* [46]. However, the leaf color conversion in pakchoi Y-05 only happens in young inner leaves (Fig. 1a), meaning that the conversion is not related with leaf senescence, but response to cold acclimation. Similarly, researchers believed that leaf color mutation is usually accompanied with blocked growth, then causing the economic losses of plants [47]. In our study, the chlorophyll content and P_n_ value in Y-05 leaves is lower than that in G-04 leaves (Fig. 1b, c), indicating that the development of chloroplasts in the yellow inner leaves of Y-05 is suppressed by cold acclimation. However, Y-05 showed well-developed phenotype (Fig. 1a). Therefore, we suggested that the outer green leaves of Y-05 may supply enough photosynthate for plants to grown. The above findings revealed that the change of inner leaf color is caused by cold acclimation, and wouldn’t affect plant development. However, why the yellow phenotype only happens in young inner leaves needs to be further investigated. One possible is that cold acclimation only plays a role in the early stage of thylakoids development in Y-05. The outer leaves, which have mature chloroplast and developed thylakoids, does not respond to cold acclimation.

Generally, low chlorophyll content is caused by inhibition of chlorophyll synthesis or chlorophyll degradation [4]. Chlorophyll synthesis includes 19 steps from the GluTR to Chl *b*, a total of 16 enzymes encoded by more than 26 genes are working in this process [48, 49]. For example, ‘White Dove’ is a leaf color mutant in kale (*Brassica oleracea*). Low temperature induced the low expression of chlorophyll biosynthesis gene *POR*, lead to chlorophyll content dramatically reduced in ‘White Dove’ [50]. *PORA*, encoding protochlorophyllide oxidoreductase, play an important role in chlorophyll biosynthesis. *Arabidopsis porA-1* seedlings suffer from a drastically reduced chlorophyll content and dwarf phenotype [51]. *OsCAO1* mainly controls the synthesis of chl *b*, its mutation will cause decreased chl *b* content, resulting in yellow-green leaf color [52]. Meanwhile, there is a dynamic balance between the biosynthesis and catabolism of chlorophyll in plants. The high expression of *CHL2* and *RCCR* genes accelerate the chlorophyll degradation, resulting in leaves yellowing in *Cymbidium sinense* [53]. Mutations in the degradation pathway of chlorophyll often lead to the phenomenon of green stagnation in plant leaves [54, 55]. Further, chlorophyll degradation in many plants is usually accompanied by senescence or injury [47]. For example, many plant show colorful leaves in autumn, which is related to chlorophyll degradation [56]. Moreover, some stresses including biotic and abiotic stress will trigger cell death and chlorophyll degradation [57]. In our study, the yellow inner leaves of Y-05 are caused by decreased chlorophyll content and undeveloped thylakoids (Fig. 1b, j, k). More important, the yellow leaves phenotype only happened in inner leaves of Y-05 but not in outer leaves. If the yellow leaves phenotype is caused by chlorophyll degradation, both outer and inner leaves should show yellow phenotype after cold acclimation. Hence, we suggested that the low chlorophyll content in yellow inner leaves of Y-05 is caused by the inhibition of chlorophyll synthesis, but not chlorophyll degradation. The idea was also supported by the impaired thylakoids membrane (Fig. 1j, k), since thylakoids membrane is the site of chlorophyll synthesis [58].

**Fig. 8:**
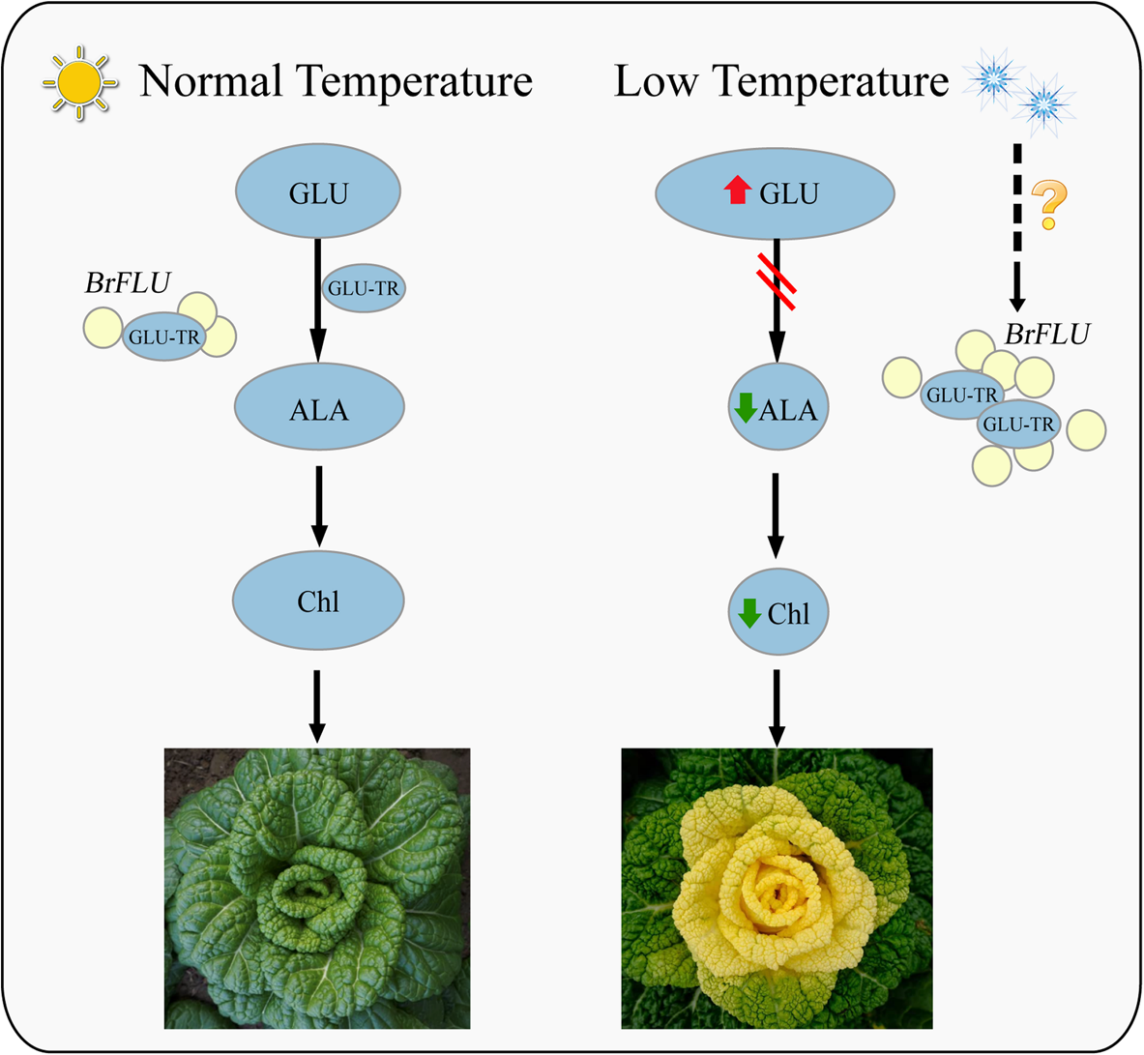
The proposed model of chlorophyll biosynthesis in Y-05 under normal or low temperature. At normal temperature, the expression of *BrFLU* remains stable, and GLU converts to ALA to maintain normal biosynthesis of chlorophyll. Under low temperature, cold acclimation can trigger an unknown regulator, inducing *BrFLU* upregulation and the interaction between BrFLU and GLU-TR to block ALA synthesis, resulting in decreased chlorophyll content and leaf yellowing in Y-05. L-Glutamic acid, glutamyl-tRNA reductase, 5-Amino-levulinate and total chlorophyll were abbreviated as Glu, GluTR, ALA and Chl, respectively. Red and green arrow represents up-regulated and down-regulated of compounds, respectively

To further investigate the mechanism of leaf color conversion in pakchoi Y-05, the transcriptomes of green outer leaves and yellow inner leaves were performed (Figs. 2, 3 and 4, Table S1–5). Both GO and KEGG analyses further confirm the low chlorophyll content (Fig. 1b), weak photosynthetic capacity (Fig. 1c) and impaired chloroplast structure (Fig. 1j, k) in inner leaves of Y-05. Metabolites serve as a bridge between genotype and phenotype. Because of metabolites are closest to phenotypes, and their changes more directly reveal gene functions [59]. Based metabolome data, we found that the low chlorophyll content in yellow inner leaves is closely associated with the block of ALA synthesis (Table S6). Complementary and inhibitory experiments of ALA (Figs. 5 and 6) further support the finding. Moreover, the transcriptomes data revealed that the high expression of *BrFLU* plays a key role in the block of ALA synthesis (Fig. S4 and Table S7). FLU protein has conserved TPR motifs at its C-terminus, which could interact with GluTR [35, 60], thus influences the rate of synthesis of ALA [41]. In previous studies on *Arabidopsis*, FLU-overexpressing lines show decreased ALA synthesis and reduced chlorophyll content in the light [60]. In our study, silencing of *BrFLU* in Y-05 pak choi further confirmed the contribution of *BrFLU* to the impaired ALA synthesis and decreased chlorophyll content (Fig. 7). Taken together, our results suggested that the impaired ALA synthesis is closely related with enhanced *BrFLU* expression, resulting in decreased chlorophyll content and leaf yellowing in Y-05. However, we did not found the significant different in the ORF and promoter sequences of *BrFLU* from Y-05 compared with other varieties (Fig. S5–6). Therefore, the reason of the specific upregulation of *BrFLU* in cold-acclimated Y-05 is not clear. In other words, the upregulation of *BrFLU* in Y-05 is regulated by an unknown regulator which responds to cold acclimation (Fig. 8). For the unknown regulator, we suggested three possibilities. One is a *BrFLU* positive transcription factor which can be induced by cold acclimation, activating *BrFLU* expression. Second is a *BrFLU* negative transcription factor which can be depressed by cold acclimation, reducing the inhibition on *BrFLU* expression. The last possibility is epigenetic regulation such as cold acclimation which may decrease *BrFLU* methylation level, resulting in enhanced *BrFLU* expression.

## Conclusions

Y-05 is a special pakchoi cultivar which shows green leaves grown under room temperature but displays yellow inner and green outer leaves after cold acclimation. In cold-acclimated Y-05 pak choi, compared with the green outer leaves, the yellow inner leaves exhibited low chlorophyll content and weak photosynthetic capacity, undeveloped chloroplasts and thylakoids. Through comprehensive analysis of transcriptome and metabonomic sequencing and functional verification, we found that cold acclimation can trigger an unknown regulator, inducing *BrFLU* upregulation to block ALA synthesis, resulting in decreased chlorophyll content and leaf yellowing (Fig. 8). However, the unknown regulator needs to be further explored. Finally, our findings provide insight into the mechanisms underlying the leaf color change response to cold acclimation.

## Methods

### Plant materials and growth conditions

All pakchoi inbred lines (Y-05, G-04, WTC, 2Q, LY, MET, SZQ) were grown in pots containing a soil: sand mixture (3: 1) in a controlled artificial climatic chamber with long-day condition (16 h light / 8 h dark) at 23 °C, 70% humidity and 250 μmol·m^− 2^·s^− 1^ light. For cold acclimation, two-month-old plants were grown 3 weeks at 4 °C, and then return to 23 °C for continues grown. After 2 weeks grown at 23 °C, cold acclimated Y-05 will exhibit green-outer leaves (GOU) and yellow-inner leaves (YIN). The GOU and YIN leaves were used for next studies. Under the same growing conditions, G-04, which showed stay-green phenotype before and after cold acclimation, was used as the control group for pigments determination and morphological observation experiments. All of the plant materials used in reseach were from State Key Laboratory of Crop Genetics and Germplasm Enhancement of Nanjing Agricultural University.

### Measurement of pigments and ALA content

The plant pigments, including chlorophyll, carotenoids contents were measured as described in previous studies [9, 61, 62] with simple modifications. In brief, 0.1 g fresh leaf was soaked in 15 ml extracting solution and then shook with 50 rpm/min for 24 h under dark condition. The absorbance was measured by an UV − vis spectrophotometer (CYTATION3, BioTek, USA) at wavelengths of 665, 649, 642, 485 and 470 nm.

$$ {\displaystyle \begin{array}{c}\mathrm{Total}\ \mathrm{Chl}=27.9\times {\mathrm{A}}_{649};\\ {}\mathrm{Chl}\ a=13.95\times {\mathrm{A}}_{665}-6.88\times {\mathrm{A}}_{649};\\ {}\begin{array}{c}\mathrm{Chl}\ b=24.96\times {\mathrm{A}}_{649}-7.32\times {\mathrm{A}}_{665};\\ {}\mathrm{Carotenoids}=\left(1000\times {\mathrm{A}}_{470}-2.05\times \mathrm{Chl}\ a+114.8\times \mathrm{Chl}\ b\right)/245.\\ {}\mathrm{Xanthophyll}=10.2\times {\mathrm{A}}_{470}-11.5\times {\mathrm{A}}_{485}-0.0036\times \mathrm{Chl}\ a-0.625\times \mathrm{Chl}\ b\end{array}\end{array}} $$

The anthocyanin was measured by method of Huo [63]. In brief, 0.1 g fresh leaf was soaked in 1 ml acidified ethanol (80% ethanol with 0.1% hydrochloric acid) and stood for 24 h under 4 °C dark condition. The absorbance was measured at wavelengths of 536 nm. All the concentrations of pigments were calculated as (mg ∙ g^− 1^). The ALA concentrations (μg ∙ g^− 1^) were measured using an enzyme-linked immunosorbent assay (ELISA) kit (Cat No: KT7958-B, Jiangsu Kete Biotechnology Co., Ltd., China).

### Transmission electron microscopy (TEM) analysis

Manufacturing methods of ultra-thin slice refer method of Maekawa [64]. In brief, Use a double-sided blade to cut 2 × 2 mm leaves from pakchoi plants and fixed them in 1% (w/v) glutaraldehyde, after washed several times with phosphate buffer, the samples were further fixed in 0.5% (w/v) osmium tetroxide. After infiltrated with resin and cut using an ultramicrotome (EM UC6, Leica Microsystems), the ultra-thin sections were obtained. For transmission electron microscopy, the ultra-thin slice was examined and photographed using a Hitachi (Tokyo, Japan) H-7650 TEM, as previously described [65].

### Transcriptome analysis

The third fully expanded leaves from the yellow part and green part from center were sampled 2 weeks after cold acclimation, the same part of three independent plants as biological replicates. After cleaned and cut, the leaf tissues were frozen in liquid nitrogen immediately, then stored in − 80 °C for further research. Total RNA was extracted from YIN and GOU of Y-05 using a TRIzaol reagent (Thermo Fisher Scientific Inc.). The library construction and RNA-seq were performed by Biomarker Technology Co. (Beijing, China). Illumina HiSeq 2500 platform (NEB, USA) was used for library preparations sequencing. After filtering (low Q-value <= 20%), cleaned reads were then assembled and mapped to the *Brassica rapa* genome (V2.5) (http://brassicadb.org/brad/index.php) using software HISAT2 (version 2.1.0) [66]. Each differentially expressed gene (DEG) function was annotated to these public databases: Nr (NCBI non-redundant protein sequences); KOG/COG (Clusters of Orthologous Groups of proteins); Pfam (Protein family), SwissProt, KEGG (Kyoto Encyclopedia of Genes and Genomes) [67] and GO (Gene Ontology). Differential expression analysis was performed using software DESeq2 [68] based on the expression levels of the genes in different samples. An absolute value of log2 (fold change) ≥2, *P*-value < 0.05 [69], as the criteria for identifying significantly differential expression.

### Untargeted metabolome analysis

The samples for untargeted metabolome analysis are same as transcriptome analysis. But to distinguish them from transcriptomes in data analysis, the metabolites in yellow-inner and green-outer leaves were named MIN and MOU respectively. Metabolite identification and quantification were performed by Biomarker Technology Co. (Beijing, China). In brief, metabolite identification was annotated against following public database: HMDB (http://www.hmdb.ca/), KEGG (Kyoto Encyclopedia of Genes and Genomes), following the standard metabolic operating procedures. Multiple-reaction monitoring (MRM) was used for metabolite quantification. Targeted UPLC-ESI-QTOF/MS profiling and multivariate data analysis (PCA and Opls-da) [70] were used to obtain more reliable information about metabolites. For all differential metabolites which has three biologically repeats, an absolute value of log2 (fold change) ≥ 1 and variable importance in projection (VIP) ≥ 1, *P*-value < 0.05 [69] were the criteria for identifying significantly differential metabolites.

### Treatment of ALA and gabaculine

For ALA treatment, once the cold acclimated Y-05 plants were transferred to 23 °C chamber, the leaves were immediately sprayed by 1 mM ALA (5-Aminolevulinic acid; CAS:5451-09-2; J&K SCIENTIFIC LTD.) solution once every 3 days for 2 weeks. The Y-05 plants sprayed by water were used as control.

For gabaculine treatment, one-month-old Y-05 seedlings without cold acclimation were watered using 50 μM gabaculine aqueous solution (3-amino-2,3-dihydrobenzoic acid; CAS: 59556–17-1, J&K SCIENTIFIC LTD, China.) solution [71] once every three days for two weeks. The Y-05 seedlings watered by water were used as control.

### Virus-induced gene silencing (VIGS) analysis

VIGS was used to generate silenced pakchoi as our previously described [72]. A specific 40-bp fragment (5′-CTGAGGAATCAAGAGCCAGAGAAGGCTTTTGAAGAGTTCATGAACTCTTCAA AAGCCTTCTCTGGCTCTTGATTCCTCAG-3′) of *BrFLU* coding region (underlined) and its antisense sequence was synthesized and inserted into the pTY vector [72]. Then, the construct was introduced into cells of one-month-old Y-05 seedlings using gene gun (Biolistic PDS-1000/He, Bio-rad, USA). The seedlings introduced by empty pTY vector were used as control. After 1 week grown under normal condition, both BrFLU-silenced and control plants were moved in 4 °C chamber for 3 weeks cold acclimation, and then return to 23 °C for continues grown.

### RNA isolation and gene expression analysis

Total RNA was extracted from leaves by using TRIzaol reagent (Thermo Fisher Scientific Inc.) [73], and cDNA was synthesized by HiScript II Q RT SuperMix for qPCR (Cat No. r223–01, Vazyme, Nanjing, China). Then analyzed by qPCR using SYBR Green Premix Pro Taq HS qPCR Kit II (Rox Plus) (Cat No. AG11719, Accurate Biotechnology (Hunan) Co.,Ltd., China) on StepOnePlus system (Applied Biosystems, USA). The relative expression of genes was analyzed by the 2^−ΔΔCT^ method [74] and were normalized to the internal control gene *BrPP2A* for pakchoi. Each reaction was performed in three technical replicates and three independent biological replicates (biological replicates: leaves of the same part of three independent plants; technical replicates: repeat detection and analysis of the same sample). Primers for qPCR analysis were designed by Primer Software Version 5.0 (Premier Biosoft International, CA, USA) and shown in Table S8.

### Data analysis

PCA of all samples and Volcano map were generated by BMK Cloud platform (www.biocloud.net). Heatmap and *cis*-acting analysis of promoter were conducted on TBtools v1.05 [75]. The full-length open reading frame (ORF) of *BrFLU* was obtained from the NCBI database using BLASTN and sequences amplified from the cultivar including Y-05, G-04, WTC, 2Q, LY, MET and SZQ. The multiple sequence alignment was used online software MultAlin [76]. The amino acid sequence alignment was used software DNAMAN. The 1.2 kb promoter sequence of *BrFLU* was identified using BLASTN to query the *B. rapa* genome. The primers were shown in Table S8. Promoter sequence analyzed using the Plant-CARE databases (http://bioinformatics.psb.ugent.be/webtools/plantcare/html/). All methods and materials in our manuscript complied with relevant institutional, national, and international guidelines and legislation.

## Supplementary Information

**Additional file 1: Table S1.** Description of read data, quality control and GC content of TIN and TOU. **Table S2.** Differential expression analysis and functional annotation of DEGs between TIN and TOU. **Table S3.** Functional categorization of DEGs between TIN and TOU by Gene ontology (GO) analysis. Table S4 Functional categorization of DEGs between TIN and TOU DEGs enriched in KEGG pathways. **Table S5.** Differential expression analysis and functional annotation of DEMs between MIN and MOU. **Table S6.** The DEMs involved in Porphyrin and chlorophyll metabolism (ko00860). **Table S7.** The expression level of *BrHEMA1*, *BrGSA1*, *BrGBP*, *BrFLU* between TIN and TOU. **Table S8.** The primers used in experiments.

**Additional file 2: Fig. S1.** The pigments content in G-04 and Y-05 leaves. **Fig. S2.** The correlation between replicates and volcano map of DEGs between TIN and TOU. **Fig. S3.** The correlation between replicates and volcano map of DEMs between MIN and MOU. **Fig. S4.** The expression profiles of *BrHEMA1*, *BrGSA1*, *BrGBP* and *BrFLU* between TIN and TOU. **Fig. S5.** Alignment of *BrFLU* nucleotide sequences in seven pakchoi varieties. **Fig. S6.** Alignment of BrFLU amino acid sequences in seven pakchoi varieties. **Fig. S7.** The promoter motif analysis of *BrFLU* in different pakchoi varieties.

## Data Availability

The datasets used and/or analysed during the current study are available from the corresponding author on reasonable request.

## Abbreviations

YIN: Yellow inner leaves
GOU: Green outer leaves
TEM: Transmission electron microscopy
DEGs: Differentially expressed genes
ALA: 5-aminolevulinic acid
*pem*: Plant etiolated mutation (*pem*)
REC: REDUCED CHLOROPLAST COVERAGE protein
TPR: Tetratricopeptide repeat protein
PPR: Pentatricopeptide repeat protein
P_n_: Net photosynthetic rate
TOU: transcripts in green outer leaves
TIN: Transcripts in yellow inner leaves
PCC: Pearson’s Correlation Coefficient
GO: Gene Ontology
KEGG: Kyoto Encyclopedia of Genes and Genomes
MIN: Metabolites in yellow inner leaves
MOU: Metabolites in green outer leaves
DEMs: Differentially expressed metabolites
Gabaculine: 3-amino 2,3-dihydrobenzoic acid
ELISA: Enzyme-linked immunosorbent assay
KOG/COG: Clusters of Orthologous Groups of proteins
Pfam: Protein family
VIGS: Virus-induced gene silencing
PCA: Principal component analysis
GluTR: Glutamyl-tRNA reductase
GSA: Glu-1-semialdehyd
GSA-AM: GSA-2,1-aminomutase
FLU: Fluorescent in blue light
FLUOE: FLU-overexpressor
GBP: GluTR-binding protein
