# Supplementary material for: Cold acclimation can specifically inhibit chlorophyll biosynthesis in young leaves of Pakchoi
Source: BMC Plant Biol. 2021 Apr 10;21:172. doi: 10.1186/s12870-021-02954-2 (PMC8035748; doi:10.1186/s12870-021-02954-2)
Supplement: Supplementary file 2 — Additional file 2: Fig. S1. The pigments content in G-04 and Y-05 leaves. Fig. S2. The correlation between replicates and volcano map of DEGs between TIN and TOU. Fig. S3. The correlation between replicates and volcano map of DEMs between MIN and MOU. Fig. S4. The expression profiles of BrHEMA1, BrGSA1, BrGBP and BrFLU between TIN and TOU. Fig. S5. Alignment of BrFLU nucleotide sequences in seven pakchoi varieties. Fig. S6. Alignment of BrFLU amino acid sequences in seven pakchoi varieties. Fig. S7. The promoter motif analysis of BrFLU in different pakchoi varieties. [file 12870_2021_2954_MOESM2_ESM.docx]

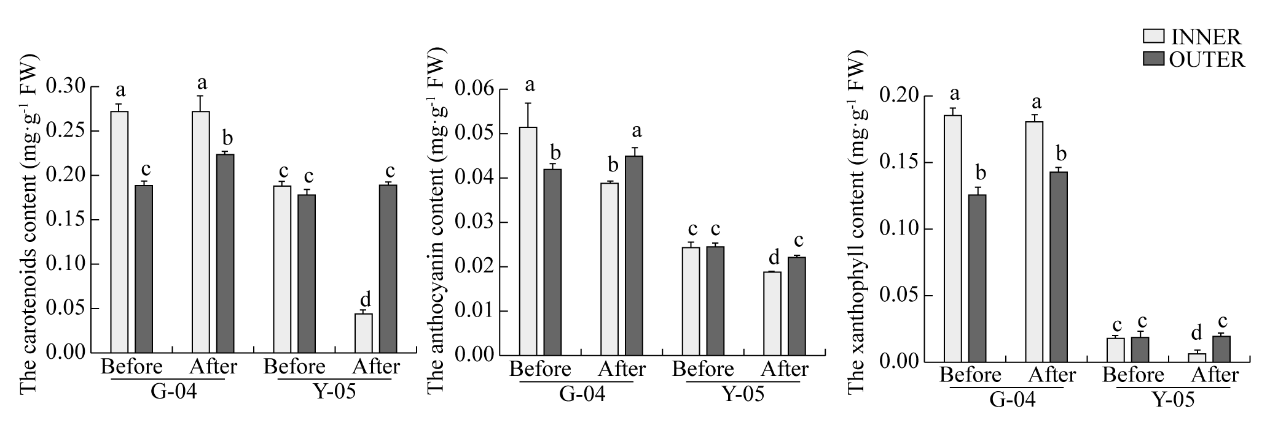


**Additional file 9: Figure S1. The pigments content in G-04 and Y-05 leaves.**

**The carotenoids****, xanthophyll and anthocyanin content of** G-04 and Y-05**. Before and after represent before and after cold acclimation, respectively. Three individual plants of each cultivar were quantified, and the pigments content were measured three times. Error bars represent SE (±SE, n=3). Different letters indicated statistically significant differences at the level of *p* < 0.05.**


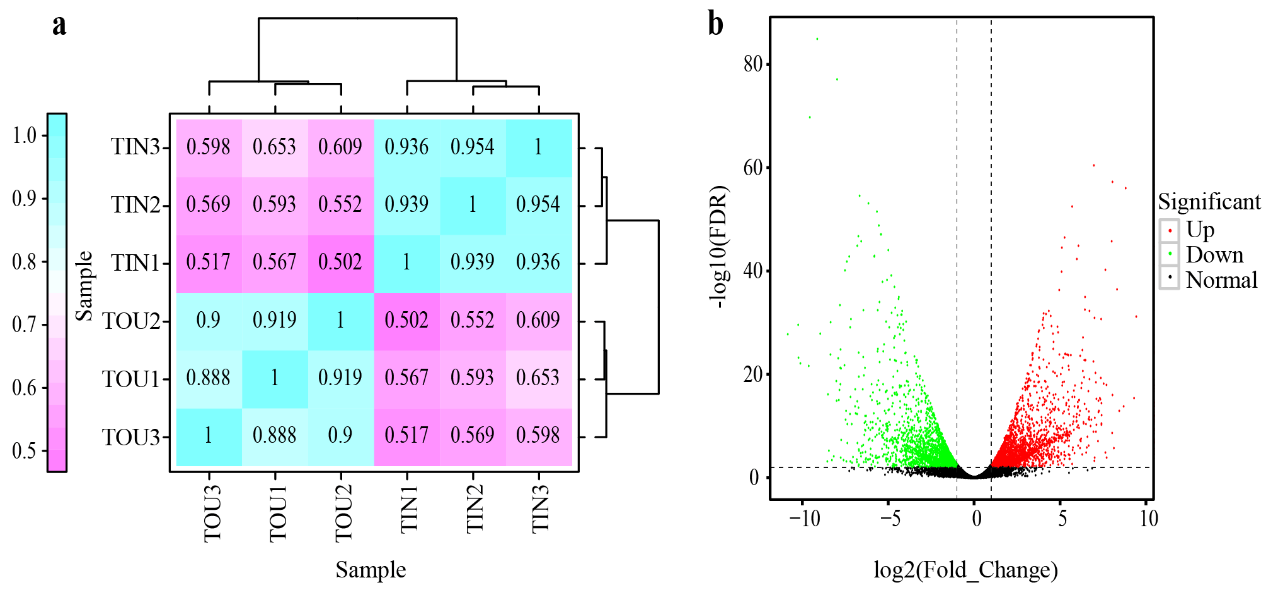


**Additional file 10: Figure S2. The correlation between replicates and volcano map of DEGs between TIN and TOU.**

(**a**) Correction coefficient between replicates. The dark color indicates the high correlation. (**b**) Volcano map of DEGs between TIN and TOU . Each point represents a gene. The X-axis represents the log_2_ (fold change) of each substance, and the Y-axis represents the -log10 (*p*-value of the student's t test), the size of the scatter points represents the VIP value of the OPLS-DA model. The green dots in the figure represent down-regulated DEGs in TIN, the red dots represent up-regulated DEG in TIN, and the black dots represent genes which were detected but not significantly different between TIN and TOU.


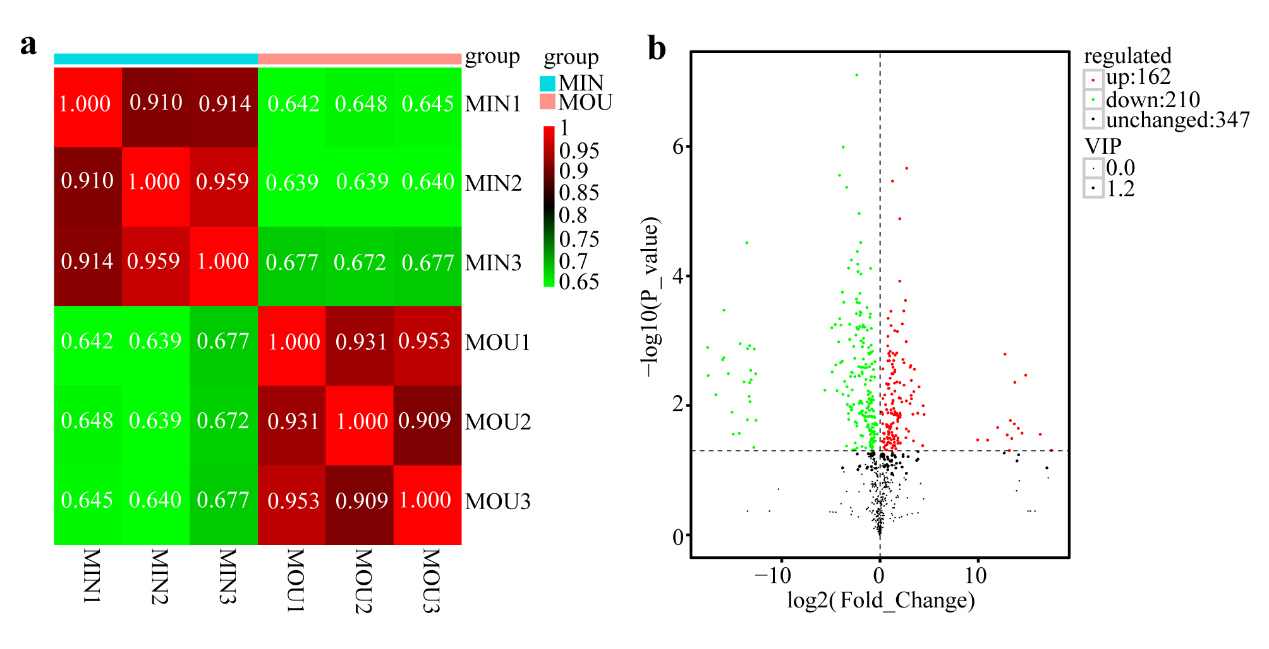


**Additional file 11: Figure S3. The correlation between replicates and volcano map of DEMs between MIN and MOU.**

(**a**) Correction coefficient between replicates. The dark color indicates the high correlation. (**b**) Volcano map of DEMs between MIN and MOU. Each point represents a metabolite. The X-axis represents the log_2_ (fold change) of each substance, and the Y-axis represents the -log10 (*p*-value of the student's t test), the size of the scatter points represents the VIP value of the OPLS-DA model. The green dots in the figure represent down-regulated DEMs in MIN, the red dots represent up-regulated DEMs in MIN, and black dots represent metabolites which were detected but not significantly different in MIN.


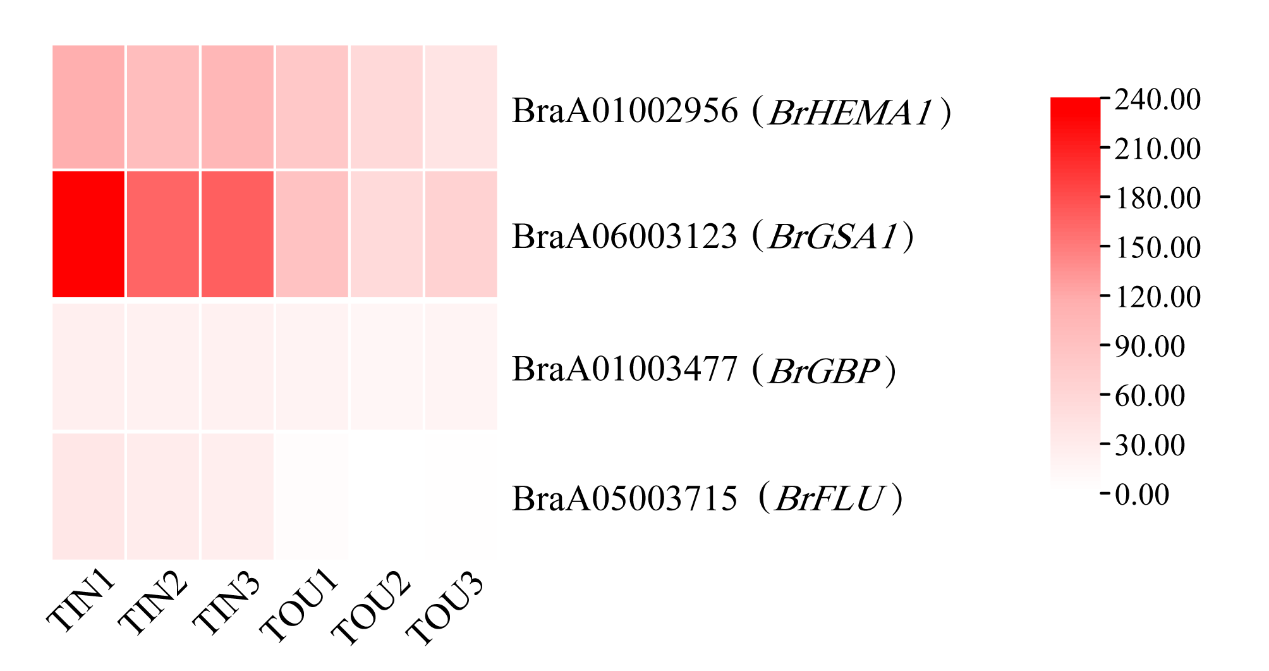


**Additional file 12: Figure S4. The expression profile of *BrHEMA1*, *BrGSA1*, *BrGBP* and *BrFLU* between TIN and TOU.**

The color scale indicates DEGs FPKM values. Each colored square represents the normalized intensity of each DEGs according to the color scale (three biological replicates × two parts, *n=6*).


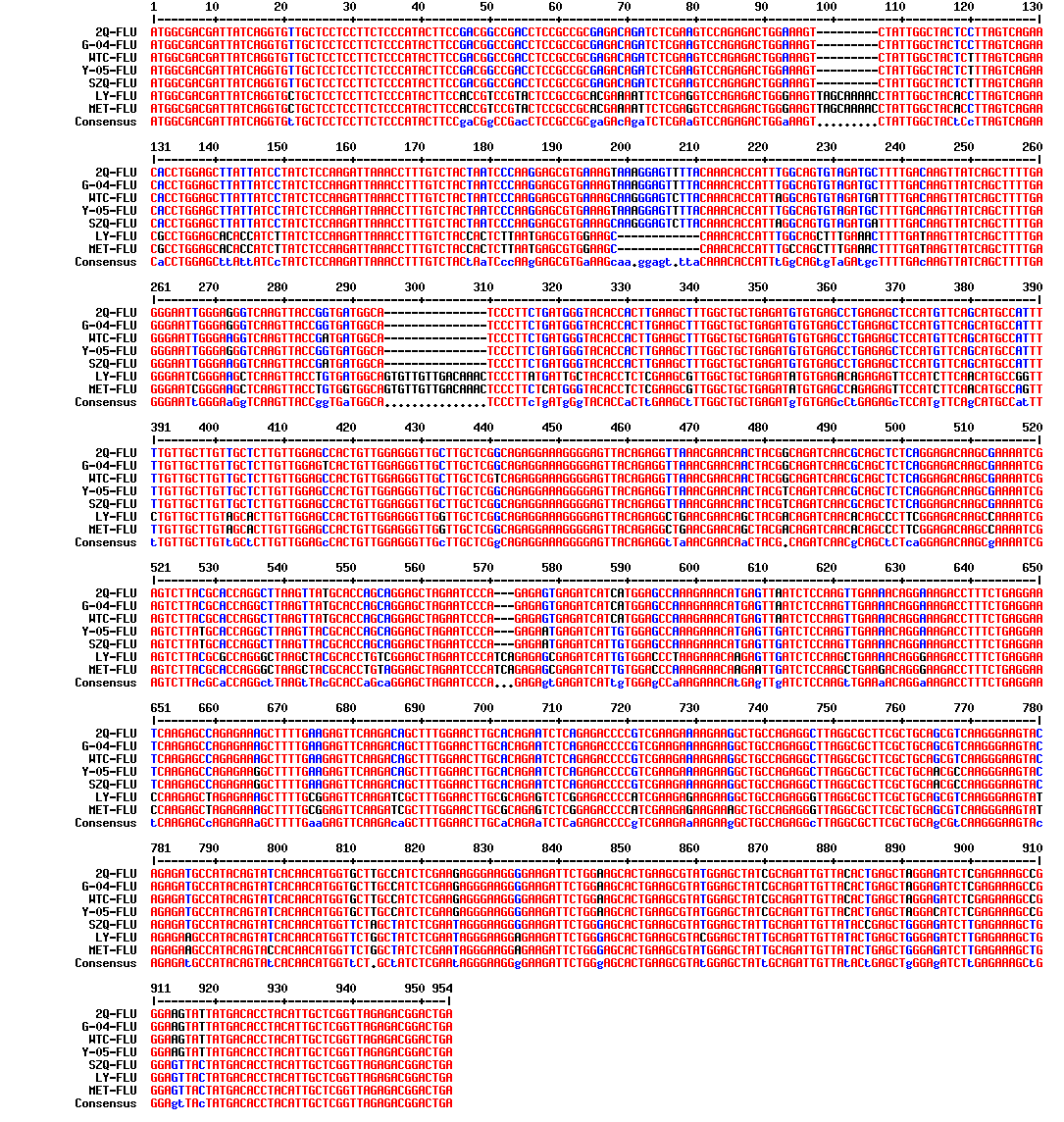


**Additional file 13: Figure S5. Alignment of *BrFLU* nucleotide sequences in seven pakchoi varieties.**

The coding sequences of *BrFLU* were obtained from Y-05 and other six pakchoi cultivars (2Q, G-04, WTC, SZQ, LY, MET). Red, black and blue letters represent high consensus, neutral and low consensus, respectively. Top line and number represent position information of nucleotide.. The dashed line is missing nucleotide sequences.


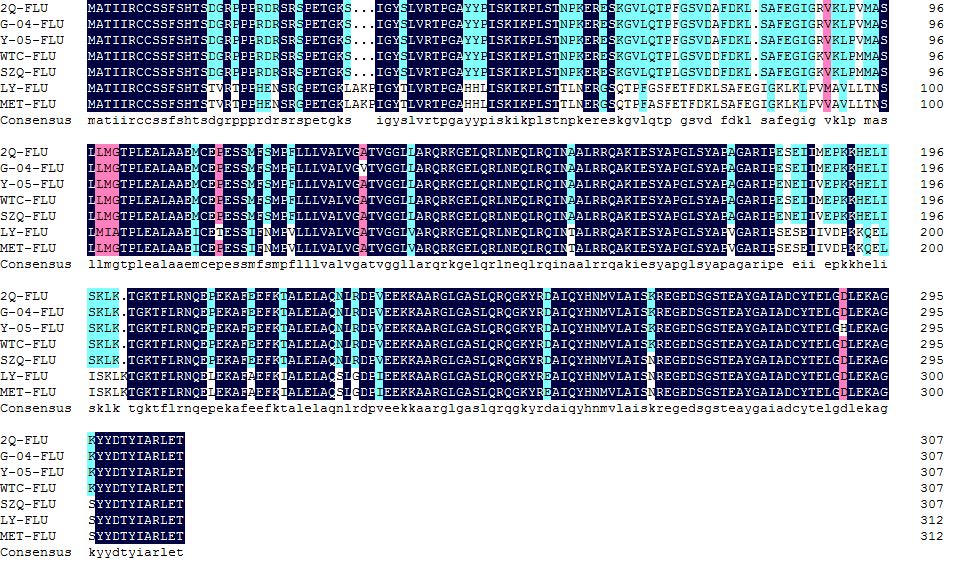


**Additional file 14: Figure S6. Alignment of BrFLU amino acid sequences in seven pakchoi varieties.**

The amino acid sequences of BrFLU were obtained from Y-05 and other six pakchoi cultivars (2Q, G-04, WTC, SZQ, LY, MET). The dotted line is missed amino acid.


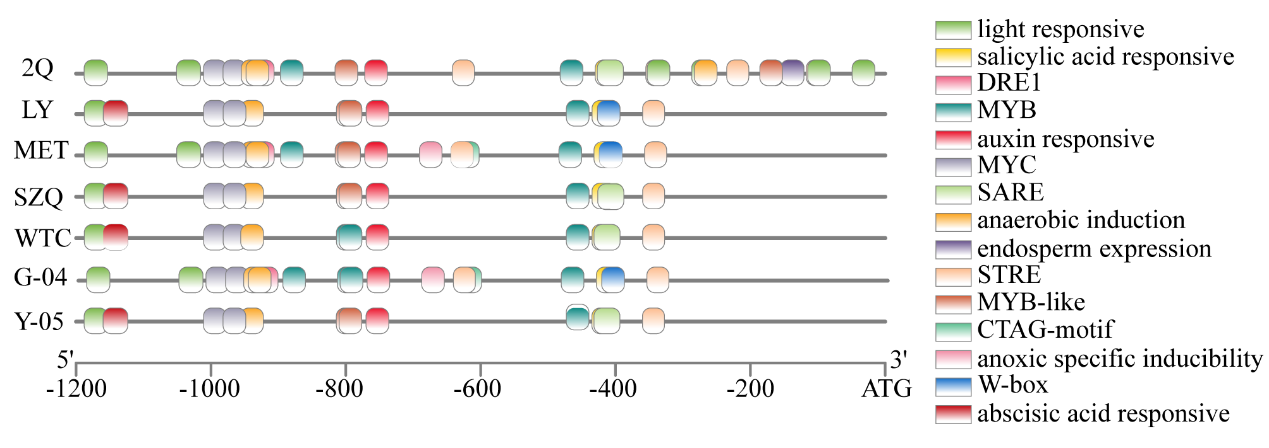


**Additional file 15: Figure S7. The promoter motif analysis of *BrFLU* in different pakchoi varieties.**

The promoter sequences of *BrFLU* were obtained from Y-05 and other six pakchoi cultivars (2Q, G-04, WTC, SZQ, LY, MET). ATG represents the initiation code of *BrFLU*. Different color square represents different motifs.
